# Preferential Binding of Lanthanides to Methanol Dehydrogenase Evaluated with Density Functional Theory

**DOI:** 10.1021/acs.jpcb.0c11077

**Published:** 2021-03-01

**Authors:** Ran Friedman

**Affiliations:** Department of Chemistry and Biomedical Sciences, Linnaeus University, Kalmar 391 82, Sweden

## Abstract

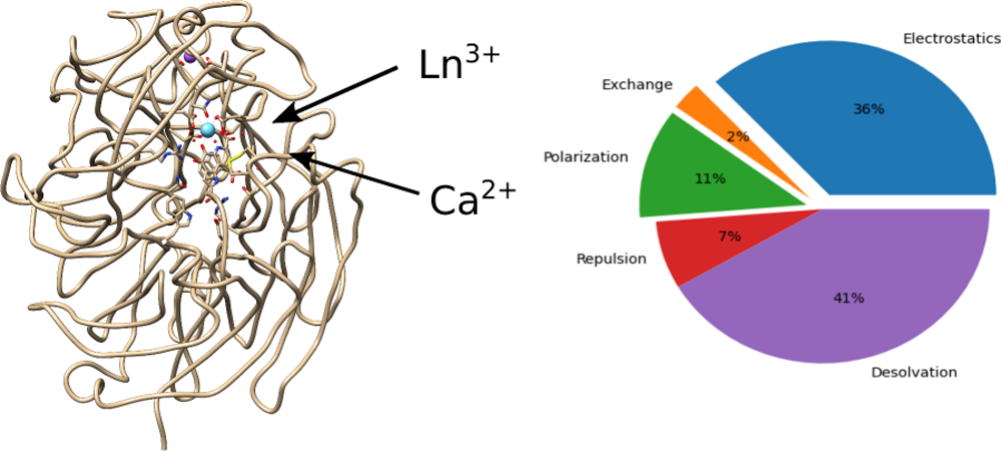

Methanol dehydrogenase (MDH) is an enzyme used by certain bacteria for the oxidation of methanol to formaldehyde, which is a necessary metabolic reaction. The discovery of a lanthanide-dependent MDH reveals that lanthanide ions (Ln^3+^) have a role in biology. Two types of MDH exist in methane-utilizing bacteria: one that is Ca^2+^-dependent (*MxaF*) and another that is Ln^3+^-dependent. Given that the triply charged Ln^3+^ are strongly hydrated, it is not clear how preference for Ln^3+^ is manifested and if the Ca^2+^-dependent *MxaF* protein can also bind Ln^3+^ ions. A computational approach was used to estimate the Gibbs energy differences between the binding of Ln^3+^ and Ca^2+^ to MDH using density functional theory. The results show that both proteins bind La^3+^ with higher affinity than Ca^2+^, albeit with a more pronounced difference in the case of Ln^3+^-dependent MDH. Interestingly, the binding of heavier lanthanides is preferred over the binding of La^3+^, with Gd^3+^ showing the highest affinity for both proteins of all Ln^3+^ ions that were tested (La^3+^, Sm^3+^, Gd^3+^, Dy^3+^, and Lu^3+^). Energy decomposition analysis reveals that the higher affinity of La^3+^ than Ca^2+^ to MDH is due to stronger contributions of electrostatics and polarization, which overcome the high cost of desolvating the ion.

## Introduction

Lanthanides are a group of metal elements with atomic numbers 57–71. Although they are often regarded as rare earth metals, lanthanides (except for the radioactive _61_Pm, which is not considered further in this article) are in fact quite abundant; even the rarest lanthanide, lutetium, is more abundant than silver and cadmium. Lanthanides are used in metallurgy, catalysis, and electronics; some lanthanide complexes have even medical uses. All lanthanides form stable Ln^3+^ ions. Some can also form Ln^4+^ or Ln^2+^ ions, but Ln^3+^ is their most stable form.

With the exception of lanthanum, the electronic configuration of the lanthanides includes one or more 4f electrons. The 6s and 5d electrons (if they exist) are shed off first, leading to Ln^3+^ ions that have 0–14 4f electrons in their outermost shell. The 4f electrons are core-like in their behavior, making all Ln^3+^ ions similar in many aspects when it comes to their chemistry. The ions tend to have hard ligands such as O and F and can adopt a large variety of configurations, with coordination numbers (CNs) that range between 2 and 12. Their similar chemistry and the fact that many lanthanides are located together in the same ore make it difficult to separate them. A major difference between the lanthanides is their ionic radius, with the heavier lanthanides having smaller radii (a phenomenon that is termed “lanthanide contraction”). This has some impact on their chemical properties as well, which can be exemplified in their hydration properties. Lighter lanthanides adopt La·(H_2_O)_9_^3+^ complexes, whereas heavier Ln^3+^ adopt hydration complexes with CN = 8. The different sizes also lead to differences in their hydration free energies, which are more favorable for the heavier lanthanides.

About a decade ago, a strain of soil bacteria was isolated in the presence of 3.0 × 10^–5^ mol dm^–3^ CeCl_3_, which could also grow in mediums containing other light lanthanide ions (La^3+^, Pr^3+^, and Nd^3+^) though not heavier ones.^1^ Later, it was established that methanotrophic bacteria (bacteria that use methane as an energy source) that were identified in volcanic mudpots grew in the presence of lanthanides as heavy as Gd^3+^ but not in mediums that contained Ca^2+^ but no Ln^3+^.^2^ Such bacteria rely on the oxidation of methanol by methanol dehydrogenase (MDH) enzymes, which were known to utilize Ca^2+^ for catalysis. Interestingly, an MDH with Ce^3+^ in its active site has been isolated, altogether suggesting that Ln^3+^ replaces Ca^2+^ as the cofactor in this enzyme.^2^ It is now widely believed that there are two types of MDH in methylotrophs, *MxaF* that relies on Ca^2+^ for catalysis and the Ln^3+^-dependent *XoxF*. Apparently, soil bacteria that grow in regions that are poor in Ca^2+^ but richer in Ln^3+^ evolved MDH that utilizes Ln^3+^ for catalysis.

Metal ions are incorporated into metalloproteins during or after folding, with Ca^2+^ ions in particular known for inducing large conformational changes upon binding.^3^ As the metal-binding sites of *MxaF* and *XoxF* are compact and highly charged, it is likely that the binding domain folds around the metal (otherwise the electrostatic repulsion will be too high). In equilibrium, the ions should bind better to the folded protein than to solvating water. Triply charged Ln^3+^ ions would theoretically bind better than Ca^2+^ to anionic binding sites in the gas phase. However, their hydration energies, which correspond to the transfer of an ion from the gas phase to water are much more favorable than the hydration energy of Ca^2+^ (Δ_hyd_*G* = −360 kcal mol^–1^ for Ca^2+^ and between −752 and −841 kcal mol^–1^ for Ln^3+^).^4^ Given that the difference in the hydration energies between Ca^2+^ and Ln^3+^ amounts to hundreds of kcal mol^–1^, it is interesting to see if *XoxF* has a preference to these ions over Ca^2+^, or if the association *MxaF*/Ca^2+^ and *XoxF*/Ln^3+^ is only owing to the difference in concentration of the ions in solution.

Here, a computational approach was used to estimate the free-energy differences between the binding of Ln^3+^ and Ca^2+^ to the protein. To this end, a model of the active site was prepared, involving the groups that are complexed to the protein.^5^ This model was then subject to density functional theory (DFT) calculations of the difference between the Gibbs binding energies between the proteins and the different ions. Benchmarking of binding energies to representative small molecules—water, acetate—CH_3_COO^–^, acetamide—CH_3_CONH_2_, and *N*-methyl-methanimine—CH_3_NHCH_3_, was made to ensure that the DFT representation is in agreement with generally accurate (yet far more expensive) coupled-cluster calculations with CCSD(T). Energy decomposition analysis with GKS-EDA was performed to shed light on the physicochemical factors that contribute to the binding of La^3+^ and Ca^2+^ to *MxaF* and *XoxF*. To examine if the preference for smaller lanthanides depends on the binding energies, the calculations of binding energies were repeated with the heaviest lanthanide ion (Lu^3+^) and Ln^3+^ ions from the middle of the series with a high-spin ground state (Sm^3+^, Gd^3+^, and Dy^3+^).

## Methods

### Benchmarking DFT Functionals

Complexes of the ions (Ca^2+^ and La^3+^) with water, acetate, acetamide, and *N*-methyl-methanimine were optimized with DFT, using the M06 functional^6^ and the def2-TZVP basis set.^7^ Effective core potential (ECP) was used for La^3+^.^8,9^ CCSD(T) reference calculations were performed at the CBS limit. The energy associated with the reaction LaL^*m*+^ + Ca^2+^ → La^3+^ + CaL^(*m*–1)+^ (see text) was used as reference. DFT calculations were performed with the def2-TZVP basis set and four functionals: B98,^10^ HSE06,^11,12^ M06,^6^ and M06-2x.^6^ All calculations were performed with NWCHEM.^13^

### Models of the Ion-Binding Sites with Ca^2+^ and Ln^3+^

The structure of *XoxF* was downloaded from the protein data bank (PDB, code 6DAM(14)). A model of the binding site was prepared by including the side chains of residues Glu^197^, Asn^185^, Asp^327^, and Asp^329^ and modeling the cofactor pyrroloquinoline quinone (PQQ) as 2ONA. The structure of *MxaF* (PDB code 1W6S(15)) was also downloaded from the PDB. Its metal-binding site model was prepared with residues Glu^177^, Asn^261^, Asp^303^, and PQQ and a water molecule. Hydrogen atoms were missing in the crystal structures and were added to the model according to the expected protonation states of the amino acid side chains using the UCSF-Chimera software.^16^ The resulting models involved 46 atoms for *XoxF* and 45 atoms for *MxaF*, including the metal.

The structures were geometry optimized while keeping the second-shell carbon atoms and the 2ONA nitrogen fixed. Geometry optimizations were carried out with the M06 DFT functional and the def2-SVPD basis set.^7,17^ ECP was used for Ln^3+^.^8,9,18^ Gibbs energies for the binding of the ions were calculated with the optimized structures (eq 1) at the M06/def2-TZVP level with additional diffuse functions on oxygen atoms and with the solvent represented by the SMD model.^19^ Calculations were performed with NWCHEM.^13^ Default atom radii for the ions were found to be inadequate for the reproduction of the solvation energies of the ions. Consequently, the radii of the ions were modified to reproduce their hydration energies^4^ in SMD calculations. The corresponding radii are given in Table 1. All calculations were performed with NWCHEM. The solvent in SMD calculations was water (with dielectric constant ϵ = 78.4). The thermal contributions owing to vibrations of the molecules could not be considered as some atoms were fixed. The calculated binding energies (eq 1) correspond to Gibbs energies owing to the inclusion of solvent contributions .^20^ Reference values for the aqueous ions were calculated in SMD directly, that is, solvation effects are implicit.

**Table 1:** Optimized Ionic Radii (Å) for Use with Ln^3+^ in SMD

Ca^2+^	La^3+^	Sm^3+^	Gd^3+^	Dy^3+^	Lu^3+^
1.822	1.963	1.858	1.830	1.804	1.757

A high-multiplicity ground state was assumed for complexes involving Sm^3+^ (sextet), Gd^3+^ (octet), and Dy^3+^ (sextet). Ligand field effects can lead to complexes with reduced multiplicity, but calculations of complexes with *XoxF* showed that the higher spin states were more stable with the basis-set used here.

### Energy Decomposition Analysis

Energy decomposition analysis was performed with GKS-EDA^21−23^ in GAMESS-US.^24^ The functional and basis set were the same as used in the binding energy calculations. Desolvation was calculated as in PCM-EDA^20^ except that in PCM-EDA (as implemented in GAMESS-US) the cavity size is the same for the complex and each component. With large systems such as the ones studied here, this results in overestimation of the binding energy (i.e., it becomes too favorable) because the solvation energies of the monomers (especially the ions) are not as favorable as they should be. To overcome this, desolvation was estimated based on the solvation energies of the complex, the free protein, and the ion as calculated for estimating the Gibbs energies of binding using NWCHEM (see above). Of note, atomic radii for the SMD model are hard-coded in GAMESS-US so that a change to the code is necessary to use the radii in Table 1, which was another reason to use NWCHEM. Adding the desolvation component from NWCHEM to GKS-EDA energies resulted in binding energies that were within 0.1 kcal mol^–1^ of those calculated with NWCHEM (Table 3), verifying that any code-dependent differences were minimal.

**Table 2:** Benchmarking of Ion–Ligand Interaction Energies^a^

ligand	Δ*E*_La–L_^int^	ΔΔ*E*_La→Ca_^int^	diff B98	diff HSE06	diff M06-2x	diff M06
water	–99.2	34.2	+1.6	+1.9	+1.5	+2.4
acetate	–527.8	194.5	–2.4	–0.8	–1.7	–1.4
acetamide	–206.0	95.6	–2.5	–1.4	–2.1	–1.8
*N*-Me-methanimine	–146.4	68.3	+0.7	+1.5	+1.6	+2.0
MAE			–0.65	+0.30	–0.17	+0.30
MUE			1.80	1.40	1.72	1.90

a Δ*E*_La–L_^int^ were calculated with CCSD(T)/CBS. ΔΔ*E*_La→Ca_^int^ values (see text) were used as references for DFT calculations. DFT calculations were performed with the def2-TZVP and different functionals. Geometry optimizations were performed using M06/def2-TZVP. All values are in kcal mol^–1^. Values represented as diff are the differences with respect to ΔΔ*E*_La→Ca_^int^ calculated with CCSD(T)/CBS. MAE—mean absolute error. MUE—mean unsigned error.

**Table 3:** Protein–Ion Interaction Energies (kcal mol^–1^)

*MxaF*—Native Cofactor Ca^2+^					
Ca^2+^	La^3+^	Sm^3+^	Gd^3+^	Dy^3+^	Lu^3+^
–42.3	–66.5	–79.3	–111.2	–80.2	–76.6

*XoxF*—Native Cofactor La^3+^					
Ca^2+^	La^3+^	Sm^3+^	Gd^3+^	Dy^3+^	Lu^3+^
–55.9	–87.3	–80.0	–138.3	–110.3	–93.7

EDA calculations were not performed for the heavier Ln^3+^ because the def2-ECP is not implemented in GAMESS-US for F core potentials and beyond. Thus, any calculations using def2-ECP for Ln^3+^ heavier than La^3+^ cannot be performed in GAMESS-US.

Graphical analysis of the EDA (table of contents figure) was performed by use of the pieplot_eda tool, available at https://github.com/Ranger1976/pieplot_eda.

## Results

### Optimized Structures of MDH Reveal Small Ion-Dependent Differences

**Figure 1:**
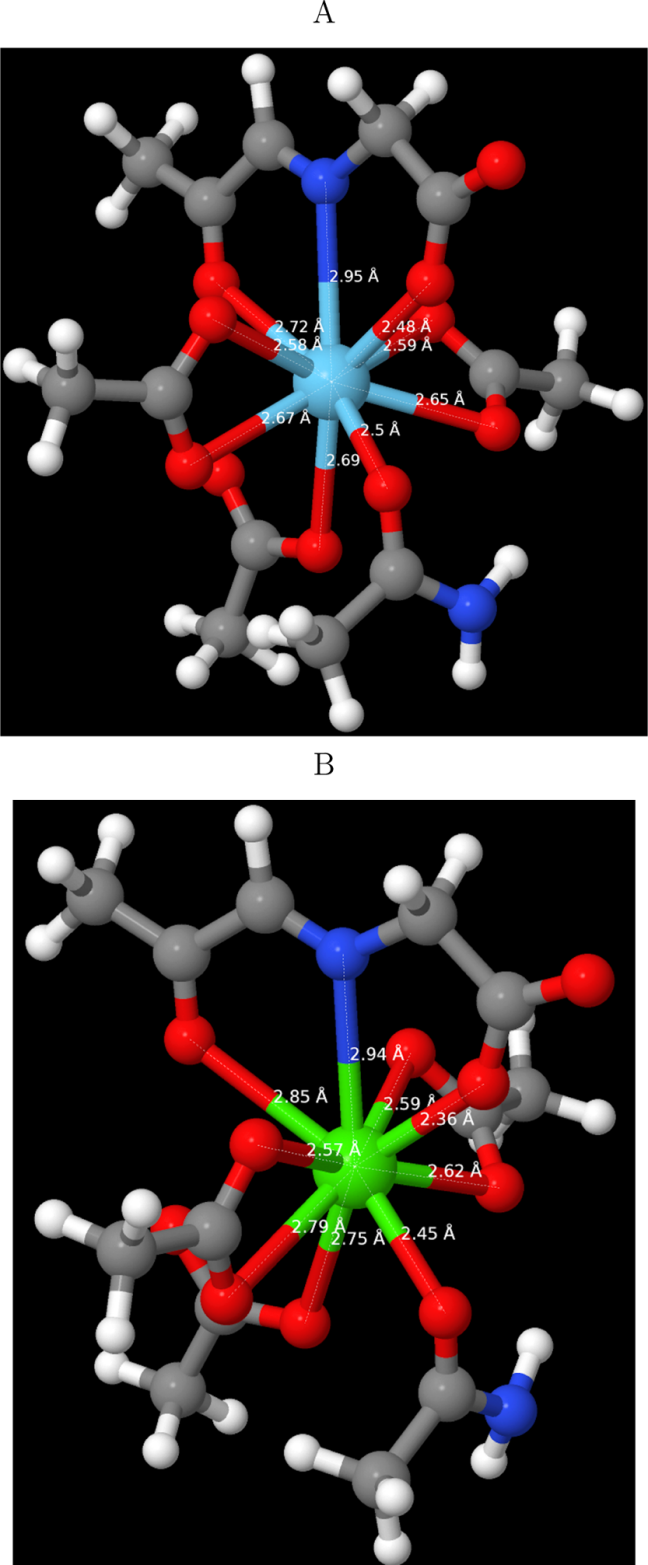
Optimized structures of the binding site of *XoxF* (PDB structure 6DAM) with (A) La^3+^ and (B) Ca^2+^.

*XoxF* binds La^3+^ through nine ligands: the two carboxylate oxygens of Glu^197^, Asn^185^:Oδ1, Asp^327^:Oδ1, the two carboxylate oxygens of Asp^329^, and three atoms of its PQQ cofactor: the quinoline nitrogen and the two oxygens most adjacent to it. To prepare a model amendable for quantum chemistry, PQQ was replaced by ((2-oxopropylidene)amino)-acetate (2oxo-*N*-Ace, 2ONA), Glu and Asp by acetates, and Asn by acetamide. After the addition of hydrogens missing in the crystal, the model was optimized with either La^3+^ or Ca^2+^ as the metal ion, keeping the second-shell carbon atoms and the 2ONA nitrogen fixed. The resulting structures (Figure 1) were highly similar. Only two metal–ligand distances varied by >0.1 Å. The distance to the 2ONA carboxylate oxygen was smaller with Ca^2+^ (2.36 Å compared to 2.48 Å), whereas the distance to the carbonyl PQQ oxygen was larger with Ca^2+^ (2.85 Å compared to 2.72 Å).

**Figure 2:**
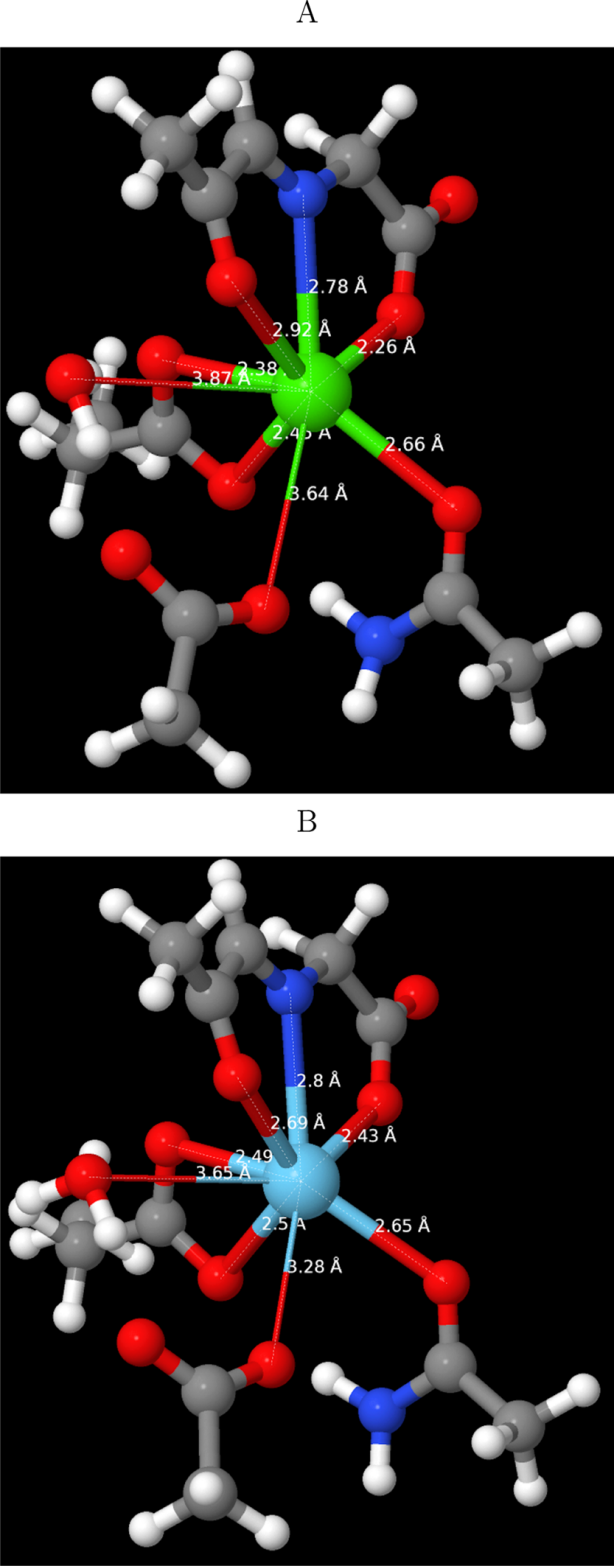
Optimized structures of the binding site of *MxaF* (PDB structure 1W6S) with (A) Ca^2+^ and (B) La^3+^.

*MxaF* binds calcium via the same three PQQ atoms as *XoxF*, the two carboxylates of Glu^177^ and Asn^261^:Oδ1 (CN = 6). Asp^303^:Oδ1 is located within 3.69 Å of the metal ion and an oxygen from a water molecule within 3.56 Å. Modeling and geometry optimizations were performed as with *XoxF* except that the glutamate was modeled as propylate. The optimized structures (Figure 2) adopted similar hexacoordinated structures, with small metal-dependent differences between the binding sites. The PQQ carboxylate oxygen was again closer to Ca^2+^ (2.26 vs 2.43 Å), and the carbonyl oxygen further away from Ca^2+^ (2.92 vs 2.69 Å). In addition, one of the glutamate oxygens was closer to Ca^2+^ (2.38 vs 2.49 Å), while the water oxygen and Asp^303^:Oδ1 were closer to La^3+^ (3.64/3.87 Å to Ca^2+^, 3.28/3.65 Å with La^3+^). These changes are in agreement with the tendency of La^3+^ to adopt structures with a higher CN.

### Different DFT Functionals Agree Well with Couple-Cluster Calculations in Calculating the Difference in Binding between Ca^2+^ and La^3+^

Although the binding site models are much smaller than the actual proteins, they are much too large to afford CCSD(T) calculations. To examine whether DFT calculations can be used to estimate the protein–ion interaction energies, benchmarking calculations were run first using the binding energies of the ions and small representative molecules: water, acetate, acetamide, and *N*-methyl-methaneimine. The reaction that was modeled was
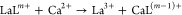
*L* is the molecular ligand in the complex. The associated potential energy change ΔΔ*E*_Ln→Ca_^int^ is expected to be positive (unfavorable) since the molecular ligands are polar molecules or anions and are thus expected to bind to La^3+^ with higher affinity.

Four DFT functionals were examined. B98^10^ (sometimes called Becke1998) is a hybrid functional that has shown good performance in a similar case.^25^ HSE06 is a range-separated functional developed by Heyd, Scuseria, and Ernzerhof.^11,12^ M06 and M06-2x are popular metahybrid functionals that differ by the amount of HF exchange.^6^ These four functionals are rather universal in their usage. While there are many other functionals that are universal in their purpose, examination of (meta)hybrid functionals that were deemed useful in a previous study of metal–protein interactions^25^ was preferred. In addition, it was desired to test if a range-separated functional could improve the results, and HSE06 was used since preliminary calculations have shown that it is rather accurate for calculations with La^3+^.

The interaction energies of the complexes with La^3+^, differences between the binding of the metals ΔΔ*E*_La→Ca_^int^, and differences between ΔΔ*E*_La→Ca_^int^ values calculated with DFT and CCSD(T) are given in Table 2. The most negative Δ*E*_La–L_^int^ value (−527.8 kcal mol^–1^) was obtained for the interaction with the acetate anion, as expected for a highly charged complex in vacuo. The least negative value was obtained for the interaction with water, which is the smallest molecule of the four. ΔΔ*E*_La→Ca_^int^ values are indeed positive and range between 34.2 kcal mol^–1^ (with water) and 194.5 kcal mol^–1^ (with acetate). The differences between the various DFT functionals and the CCSD(T) reference were small in all cases, with mean unsigned errors (MUE) of 1.40–1.90 kcal/mol. Given the small variation between the four functionals, they were all deemed equally valid and further calculations were performed with M06. The HSE06 functional had the smallest MUE but is more demanding computationally.

### *MxaF* and *XoxF* Bind Better to La^3+^ than to Ca^2+^

After verifying that DFT calculations are rather accurate for the model molecules, binding energies between each of the proteins and the two ions, La^3+^ and Ca^2+^ were calculated as
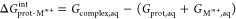
1where *G*_complex,aq_, *G*_prot,aq_, and *G*_M^*m*+^,aq_ are the Gibbs energies of the complex, protein binding site, and metal ion, respectively, when solvated in water. Gibbs energies were calculated at the M06/def2-TZVP level with additional diffuse functions on oxygen atoms and with the solvent (water) represented by the SMD model.^19^

The fact that the interaction energies with model compounds were more favorable for La^3+^ (Table 2) does not necessarily mean that the Gibbs interactions energies are more favorable for the same ion since the triply charged Ln^3+^ ions have much more negative hydration energies and hence *G*_M^*m*+^,aq_ is lower. However, when comparing Ca^2+^ and La^3+^, it can be seen that Δ*G*_prot-M^*m*+^_^int^ is more negative with La^3+^ as the bound ion in both *MxaF* and *XoxF*. The preference for La^3+^ is more pronounced in *XoxF* that is a Ln^3+^-dependent MDH than in the Ca^2+^-dependent MDH, which suggests that the evolution of *XoxF* selected for a conformation is even more likely to bind Ln^3+^ ions (the difference is 31.4 and 24.2 kcal mol^–1^ in *XoxF* and *MxaF*, respectively).

### Energy Decomposition Analysis Reveals Differences in the Contributions to Binding of Ca^2+^ and La^3+^ to MDH

To gain a better insight into the proteins binding La^3+^ better than Ca^2+^ despite the more negative hydration energy of the former, energy decomposition analysis was performed with GKS-EDA.^22,23^ This analysis, when performed with *XoxF* (Table 4), revealed that the highest contribution was the desolvation energy which opposes binding since both the ions and the negatively charged binding site lose favorable interactions with the water. The cost of desolvation was much higher for the conformation that binds La^3+^. The difference in hydration energies (392 kcal mol^–1^) between the ions does not account for the difference in Gibbs desolvation energies upon complexation (523 kcal mol^–1^). This reveals that the small differences between the structures have an associated effect on binding. As expected for a highly charged complex, electrostatics dominated the favorable interactions and accounted for 79 and 72% of the favorable contributions when Ca^2+^ and La^3+^, respectively, bind to *XoxF*. Polarization was also significant, more so in the binding of La^3+^. Exchange accounted for about 5% of the favorable interactions. The results were qualitatively similar for *MxaF* (Table 5).

**Table 4:** Energy Decomposition Analysis for *XoxF*–Ion Interactions^a^

contribution	Ca^2+^	La^3+^
electrostatics	–837.14	–1254.45
exchange	–37.85	–98.65
repulsion	96.78	241.83
polarization	–180.70	–389.85
correlation	12.82	1.05
desolvation	890.15	1412.88
all favorable	–1055.69	–1742.95
electrostatic (%)	79	72
polarization (%)	17	22
exchange (%)	4	6

a All values in kcal mol^–1^.

**Table 5:** Energy Decomposition Analysis for *MxaF*–Ion Interactions^a^

contribution	Ca^2+^	La^3+^
electrostatics	–649.48	–979.74
exchange	–42.81	–86.37
repulsion	109.70	212.23
polarization	–151.32	–332.83
correlation	0.41	–11.08
desolvation	691.19	1131.01
all favorable	–843.59	–1409.92
electrostatic (%)	77	69
polarization (%)	18	24
exchange (%)	5	6
correlation (%)		1

a All values in kcal mol ^–1^.

### Heavier Lanthanides, Especially Gd^3+^, Bind MDH Better than La^3+^

Given that La^3+^ binds the two different MDH better than Ca^2+^, it is interesting to examine whether there is a preference for certain Ln^3+^ ions (Table 3). The heavier is a Ln^3+^ ion, smaller and more strongly hydrated it is. The binding of the heaviest Ln^3+^, Lu^3+^ was tested first, and the ion was shown to bind both MDH proteins more strongly than La^3+^ (by 10.1 and 6.4 kcal/mol in *MxaF* and *XoxF*, respectively).

The binding of three additional Ln^3+^ ions was examined next: Sm^3+^, Gd^3+^, and Dy^3+^. These are ions from the middle of the lanthanide series that are characterized by high-spin ground states (sextet for Sm^3+^ and Dy^3+^, octet for Gd^3+^). Interestingly, Gd^3+^ bound both proteins with an affinity much higher than the other Ln^3+^ ions. Considering *MxaF*, Sm^3+^ and Dy^3+^ had similar binding affinities to the protein, 3–4 kcal mol^–1^ stronger than Lu^3+^. Dy^3+^ (but not Sm^3+^) bound better than Lu^3+^ also to *XoxF*.

## Discussion

The two MDH (*MxaF* and *XoxF*) operate with the same PQQ cofactor but with different metal ions in their catalytic sites. Using DFT calculations, it was shown here that both proteins can bind Ca^2+^ and different Ln^3+^ ions and that the Ln^3+^ ions bind with better affinity. The chemical properties of the Ln^3+^ are very similar; they have a small but persistent difference in their ionic size that also leads to the smaller and heavier ions being more strongly hydrated despite binding fewer water molecules. Both proteins were shown to bind Ln^3+^ ions, light and heavy, better than Ca^2+^. When it comes to binding affinities to *MxaF* and *XoxF*, there is a stronger preference for La^3+^ over Ca^2+^ in *XoxF*. Differences were found between the structures of the Ca^2+^-bound and La^3+^-bound proteins, but these are not pronounced and the coordination is almost the same when they bind Ca^2+^ and La^3+^.

One limitation of the models used here is their small size. It is possible that second and higher shell residues could affect Δ*G*_prot-M^*m*+^_^int^ and the metal selectivity. Interestingly, there is a similarity in the binding sites when it comes to second-shell residues (Table 6). In both cases, a Trp residue is hydrogen bonded to Glu in the first shell; whereas Asp residue(s) in the first shell are neutralized by Arg and hydrogen bond to a Trp. In principle, QM/MM calculations could be used to analyze long-term interactions. Whereas such calculations are well-suited for, for example, enzymatic reactions, their suitability for the estimation of Gibbs energies of interactions between a protein and an ion or a small molecule is limited. Such calculations require careful consideration of the bulk solvent. Explicit treatment of the solvent is computationally intractable because, at the very least, 10^3^ to 10^4^ water molecules should be considered. In implicit solvent treatments, different approximations are used for the MM and QM parts. As a consequence, while the underlying theory affords the use of QM/MM calculations for systems such as the one considered here, such calculations are not practical for Δ*G*_prot-M^*m*+^_^int^.

**Table 6:** First- and Second-Shell Residues That Bind to the Metal Ions in *MxaF* and *XoxF*^a^

*MxaF*/Ca^2+^		*XoxF*/La^3+^	
first shell	second shell	first shell	second shell
Glu^177^	Trp^265^	Glu^197^	Trp^289^, w1035, w1062
Asn^261^		Asn^285^	w1041
Asp^303^	Trp^265^, Arg^331^	Asp^327^	Arg^354^, w834
		Asp^329^	Trp^267^, Arg^354^
PQQ	Thr^159^, Ser^174^	PQQ^b^	Gly^196^, w1041

a Residues that can form hydrogen bonds with the metal-ion ligands are considered as the second shell. “w” stands for water.

b Residue Thr^265^ binds to the distal oxygen of the PQQ carboxylate, which is not directly coordinated to the metal. The charge of the coordination shell and types of ligands both affect the affinity for ions when a native metal cofactor is replaced.^26,27^

Calculations have shown that the maximum number of carboxylate ligands in the binding site of a metal M^*m*+^ is *m* + 1.^28^ This seems to agree well with the native binding shells of *MxaF* and *XoxF* and can explain why preference for Ln^3+^ is more pronounced in the binding site of *XoxF* that includes four such ligands (Glu^197^, Asp^327^, Asp^329^, and PQQ). The binding site might be even more negative if a second-shell residue forms a salt bridge with one of the carboxylates,^27^ which explains why Ca^3+^ still binds well to the binding site of *XoxF*. The solvated ions and protein were considered here as the reference state, and hence reference calculations were performed in water. When considering the protein environment, other, less polar solvents are often used as references. Indeed, in a study considering the binding of cadmium (and other ions) to proteins, the solvated complexes were studied in water and tetrahydrofuran (THF, ϵ = 7.58). However, differences in the binding energies (Δ*G*_prot-M^*m*+^_^int^ compared to either water or THF) were rather small.^5^ Interestingly, in a study of a binding of a charged drug to a protein, the Gibbs energy of binding was found to be more accurate when the reference was water.^29^ Considering binding of singly charged ions on the protein surface, the electrostatic contribution to the Gibbs energy was in line with the total free energy when the dielectric constant was higher (ϵ = 40) rather than lower (ϵ = 10 or 4).^30^

The hydration energies of lanthanides and their stability constants when binding to polyaminocarboxylate ligands^31^ vary monotonously with the atomic number. The situation is different with the two MDH proteins, with the binding energy (in absolute value, and hence stability constant) being highest for Gd^3+^. There is apparently some complex trade-off between the fully hydrated Ln^3+^ and the protein-bound one, with Gd^3+^, in the middle of the series, showing the highest stability in the complex. Lanthanides are notoriously difficult to separate, and the finding that the series of stability constants in proteins is different than the hydration energies or stability constants for chelators might motivate the development of more specific separation methods or better chelators in case of accidental exposure of human or animals to Ln^3+^.

EDA calculations revealed the dominance of desolvation and electrostatics in the binding of Ca^2+^ and La^3+^ to MDH. The polarization term accounts for a larger share of the binding energy for the triply-charged La^3+^, although the ion is rather hard. It was not possible to run similar calculations with the heavier lanthanides because such calculations are not possible with the core potentials of Ln^3+^ ions in GAMESS-US using the def2-series of basis sets. Given that the F-electrons do not participate in binding, it might be expected that the difference between other Ln^3+^ and Ca^2+^ will be of the same nature.

Given the higher affinity of the proteins to Lu^3+^ than to La^3+^, it may be possible that MDH or other proteins that bind to Ln^3+^ heavier than Gd^3+^ will be identified. However, for such ions to be operative, they must gain access to the cells through active transport over the cell membrane, and no suitable transport protein has been identified. In addition, the heavier lanthanides are overall less common and lighter lanthanides will normally be taken up sooner. Elucidation of the transport mechanism of Ln^3+^ ions into cells will be of high interest, not only for microbiology but also for a better understanding of how these ions interact with proteins in case of exposure to Ln salts.
